# Long‐Term Persistence of Hepatitis A Virus Immunity in Healthcare Workers Upto 25 Years After Vaccination

**DOI:** 10.1111/jvh.70187

**Published:** 2026-05-12

**Authors:** Chiara Noviello, Maurizio Aricò, Francesco Livio Scazzi, Giacomo Riformato, Onofrio Florio, Alessandro Furio, Angela Maria Vittoria Larocca, Claudia Palmieri, Stefano Manzi, Michele Alberto Cantalice, Silvio Tafuri, Pasquale Stefanizzi

**Affiliations:** ^1^ Interdisciplinary Department of Medicine Aldo Moro University of Bari Bari Italy; ^2^ Control Room Unit Bari Policlinico General Hospital Bari Italy

**Keywords:** combined HAV/HBV vaccine, healthcare workers, hepatitis a virus, long‐term immunity, vaccine durability

## Abstract

Hepatitis A virus (HAV) remains globally endemic, particularly in populations with limited sanitation and poses risks to travellers and healthcare personnel. Although vaccination provides long‐term protection, data on the duration of immunity in occupationally exposed groups are limited. We conducted a prospective cohort study among healthcare personnel and medical students vaccinated with either a monovalent HAV vaccine (Havrix, Avaxim) or a combined HAV/HBV vaccine (Twinrix) between 1996 and 2003 at Bari University Hospital, Italy. Vaccination records were verified, and serum anti‐HAV IgG concentrations were quantified using CMIA (ARCHITECT HAVAb‐IgG). Statistical analyses included Student's *t*‐test, *χ* ^2^ test and logistic regression (STATA MP 11). A total of 334 participants were included (167 per group; mean age 31 vs. 36 years). After a median of 23 years since vaccination, seroprotection rates were 94.6% for the monovalent and 97.0% for the bivalent vaccine groups (*p* = 0.28). Mean anti‐HAV IgG concentrations were significantly higher in the bivalent vaccine group (10.9 ± 4.6 IU/mL) than in the monovalent group (8.3 ± 4.6 IU/mL; *p* < 0.001). No associations were observed with sex or behavioural factors, and younger age was the only independent predictor of higher antibody levels (*β* = 0.32; 95% CI, 0.10–0.43; *p* < 0.001). These findings demonstrate that protective HAV antibody levels persist for more than two decades after vaccination. Both monovalent and bivalent vaccines confer durable immunity, supporting the continued inclusion of HAV vaccination in national immunisation programs and occupational health strategies.

## 1 Background

Hepatitis A virus (HAV) is an enterically transmitted pathogen with worldwide distribution, responsible for approximately 1.5 million human infections and 39,000 deaths in 2019 [1]. The virus demonstrates remarkable environmental resilience [2, 3], remaining viable for extended periods on surfaces, skin, food products and in sewage. Transmission occurs primarily via the faecal–oral route [4], typically through contaminated food or water. Although no specific antiviral therapy exists [5], infection confers lifelong immunity in recovered individuals [6].

Prevention relies on vaccination and environmental control measures, including improved hygiene, safe food handling, access to clean water and appropriate wastewater management [5]. The global burden of acute hepatitis A (HA) correlates strongly with socioeconomic development [7]. Low‐ and middle‐income countries, particularly in Africa and South Asia, continue to report the highest incidence [8]. In 1990, approximately 80% of cases occurred in individuals under 15 years of age, exceeding 90% in Africa. By 2019, the mean age at infection had increased, yet children remained the most affected group in low‐income settings, whereas adult infections are increasingly reported in high‐income regions [8]. Globalisation, travel and migration have reshaped acute hepatitis A epidemiology [8], introducing new risks among vulnerable groups, including men who have sex with men (MSM) [9], immunocompromised individuals, homeless persons and people who use drugs [8].

In the European Union/European Economic Area (EU/EEA), HA was the fifth most frequently reported infectious disease in 2022 [10], with 4548 notified cases (rate: 1 per 100,000 population). Children aged 5–14 years accounted for 20% of cases and had the highest notification rate, reflecting ongoing transmission in younger age groups. However, despite this higher incidence in children, the relative proportion of adult cases has increased in recent years. This trend is consistent with the epidemiological shift observed in low‐endemicity settings, where improved sanitation reduces early‐life exposure and leads to an accumulation of susceptible individuals into adulthood [11]. As a result, infections are increasingly observed among adults, who are at higher risk of severe disease, hospitalisation and death [10]. Between January and May 2025, Austria, Czechia, Hungary and Slovakia reported an unexpected increase in HAV subgenotype IB infections, mainly affecting marginalised and Roma communities [12], highlighting persistent social and environmental inequalities.

In Italy, 443 HA cases were reported in 2024, mainly in central and northern regions, with an incidence increase from 0.51 per 100,000 in 2023 to 0.85 in 2024. Foodborne transmission remains predominant, particularly through raw or undercooked shellfish and frozen mixed berries. Nearly half of the cases occurred among individuals for whom vaccination is recommended, including travellers to endemic areas, MSM and contacts of infected persons, indicating missed prevention opportunities [13].

The Apulia region historically exhibited high HA endemicity. A large outbreak in 1996–1997 [14] resulted in approximately 11,000 reported cases [15], imposing a substantial healthcare burden [16]. Subsequently, a regional vaccination program targeting infants (15–18 months) and adolescents (12 years) was implemented, substantially reducing HAV incidence.

Infants were immunised using the monovalent HAV inactivated vaccines, while for adolescents bivalent HAV/HBV vaccine was routinely adopted, using the catch‐up campaign for HBV as opportune occasion for HAV vaccine administration. The use of bivalent HAV/HBV vaccines during the hepatitis B catch‐up campaign contributed to high coverage and herd immunity. The routinely use of bivalent HAV/HBV vaccine for catch‐up campaign stopped in 2003 [17]. According to international guidelines and manufacturer recommendations, the monovalent HAV vaccine is administered in a two‐dose schedule (0 and 6–12 months), whereas the combined HAV/HBV vaccine follows a three‐dose schedule (0, 1 and 6 months) [18].

Nevertheless, due to traditional dietary habits, particularly raw seafood consumption, Apulia remains an area of residual exposure risk. Serological studies indicate that individuals aged under 30 years exhibit lower anti‐HAV seroprevalence and higher susceptibility to infection [19].

Inactivated HAV vaccines—both monovalent and combined HAV/HBV formulations—are reported as highly effective. Population‐based studies from Australia, China, Israel, Panama and the United States demonstrated 76%–98% reductions in incidence following vaccine introduction, particularly among children [20]. In Argentina, adoption of a single‐dose schedule led to marked declines in symptomatic and fulminant HA, as well as a reduction in liver transplantation rates [21]. Nevertheless, concurrent improvements in sanitation and hygiene have also contributed to these declines, emphasising the combined role of vaccination and environmental interventions in HAV control [1].

Vaccine‐induced protection against HAV is known to persist for at least 15 years [22].

However, others' long‐term follow‐up and mathematical modelling studies suggest persistence of protective immunity for 20–30 years or longer, potentially lifelong in immunocompetent individuals [23, 24]. Despite this long‐term data in European populations, particularly among healthcare workers (HCWs), a population potentially at occupational risk of exposure, remains limited. Most occupational immunisation programs focus on hepatitis B [25] leaving uncertainty about the duration of HAV immunity in healthcare settings. As HCWs may be exposed to HAV through patient care, laboratory work or environmental contamination, understanding the longevity of vaccine‐induced protection is essential for a tailored occupational health policy.

The present study aims to evaluate real life long‐term immunogenicity of the HAV vaccine among HCWs of a third level, teaching hospital in Apulia, who completed vaccination between 1996 and 2003 during a catch‐up universal mass vaccination campaign. Specifically, it investigates: (1) the persistence of protective anti‐HAV IgG levels up to nearly three decades post‐vaccination; and (2) potential differences in long‐term immune response between monovalent and bivalent HAV vaccine formulations. These findings are expected to inform evidence‐based vaccination strategies and occupational health recommendations regarding HAV immunisation in healthcare professionals.

## 2 Methods

### 2.1 Study Design and Population

This prospective cohort study evaluated the long‐term immunogenicity of hepatitis A vaccination by comparing cohorts of individuals who had received different vaccine formulations. The study assessed immunological outcomes among HCWs and medical students who had previously completed a full primary vaccination schedule with either a monovalent HAV vaccine (*Havrix* ‐GlaxoSmithKline, Brentford, UK‐ or *Avaxim* ‐Sanofi Pasteur, Lyon, France) or a bivalent HAV/HBV vaccine (*Twinrix* GlaxoSmithKline, Brentford, United Kingdom) between 1996 and 2003, during a catch‐up universal mass vaccination campaign. The target population comprised healthcare personnel and medical students at the third‐level teaching hospital and medical school in Bari, Italy. Both were evaluated as part of their routine health surveillance program. Participants were recruited using a convenience sampling approach based on accessibility and institutional affiliation.

### 2.2 Eligibility Criteria

Participants were eligible if they had received a complete primary HAV vaccination series (monovalent or bivalent formulation) between 1996 and 2003 and if detailed documentation of vaccination and subsequent serological testing was available.

A complete primary vaccination schedule was defined as follows: for the monovalent inactivated HAV vaccine (Havrix), two doses administered at least 6 months apart (up to a maximum interval of 12 months); for the combined HAV/HBV vaccine, three doses administered at 0, 1 and 6 months, regardless of age.

Eligible individuals were required to be in good health at the time of sample collection or to have medical conditions predefined as acceptable by the study protocol.

Exclusion criteria included any of the following: immunodeficiency or ongoing immunosuppressive therapy at the time of vaccination; vaccination outside the defined time window (before 1996 or after 2003); incomplete vaccination schedule or missing documentation; history of clinically confirmed HAV infection; refusal to provide written consent.

### 2.3 Vaccination Verification and Data Collection

Vaccination status was verified through the Regional Digital Immunization Registry (GIAVA) and/or medical records provided by participants.

Participants completed a standardised questionnaire collecting data on prior HAV infection, vaccination history, dietary habits (including consumption of raw seafood), travel to endemic areas and medical conditions potentially influencing immune response.

### 2.4 Serological Testing

For each participant, a 5‐mL venous blood sample was collected and analysed. Serum anti‐HAV IgG concentrations were quantified using the ARCHITECT HAVAb‐IgG chemiluminescent microparticle immunoassay (CMIA; Abbott Diagnostics, Chicago, IL, USA). Results were expressed as the ratio of sample relative light units (RLU) to assay cut‐off (CO) values (S/CO). Samples with S/CO ≥ 1.0 were classified as reactive (seropositive). Seroprotection was defined as anti‐HAV IgG levels ≥ 1.0 S/CO, corresponding to assay‐defined reactivity and considered indicative of protective immunity.

### 2.5 Sample Size Estimation

Based on previous serosurveillance data indicating a 90% prevalence of protective antibody levels [14] a minimum of 139 participants per vaccine group was required to achieve 95% confidence with a 5% standard error. To accommodate potential indeterminate or missing results, the target enrolment was increased to 167 participants per group.

### 2.6 Statistical Analysis

All statistical analyses were performed using STATA MP 11 (StataCorp, College Station, TX, USA). Continuous variables were expressed as geometric means concentrations (GMCs) and, for descriptive purposes, as means ± standard deviations (SDs) and categorical variables were reported as proportions with 95% confidence intervals (CIs).

Comparisons of demographic characteristics (e.g., gender, age) and immunological outcomes between vaccine groups were performed using the Student's *t*‐test for continuous variables and the chi‐squared test for categorical variables. For non‐normally distributed variables, appropriate non‐parametric tests were applied.

To identify determinants of seroprotection (defined as anti‐HAV IgG levels ≥ 1.0 S/CO following primary vaccination), univariate logistic regression was applied, with seroprotection status as the dependent variable and vaccine type, sex, age, vaccination interval and time since vaccination as predictors. Odds ratios (ORs) with 95% CIs were reported.

Variables with *p* < 0.05 in univariate analysis were included in a multivariate logistic regression model, adjusted odds ratios (aORs) with 95% Cis were calculated.

Associations between IgG titres and questionnaire‐based variables were analysed using non‐parametric tests: the Mann–Whitney *U* test for binary variables and the Kruskal–Wallis test for categorical variables with ≥ 3 levels.

All statistical tests were two‐sided, and *p* < 0.05 was considered statistically significant.

### 2.7 Ethics Statement

The study protocol was approved by the Local Ethics Committee of the Azienda Ospedaliero‐Universitaria Consorziale Policlinico di Bari on September 30, 2024 (Approval No. 1883/CEL), in accordance with the Declaration of Helsinki. Written informed consent was obtained from all participants prior to study enrolment.

## 3 Results

A total of 334 HCWs were enrolled in the study, including 167 vaccinated with the bivalent HAV/HBV vaccine and 167 vaccinated with the monovalent HAV vaccine. In the bivalent group, 100 participants were female (59.9%) and 67 were male (40.1%), with an average age of 36 years and a mean interval since last vaccination of 23.7 ± 1.5 years (median 23.5). In the monovalent group, 98 were female (58.7%) and 69 male (41.3%), with a mean age of 31 years and mean time since vaccination of 24.0 ± 2.6 years (median 23.8).

The two groups were comparable in terms of gender distribution (*χ* ^2^ = 0.012; df = 1; *p* = 0.91) and years elapsed since the last vaccination (*t* = 1.06; *p* = 0.28). Conversely, a statistically significant difference was observed in average age (*t* = −13.64; *p* < 0.01), with participants in the group vaccinated against hepatitis A+B being older on average than those vaccinated against hepatitis A.

Among participants receiving the bivalent vaccine, 162 (97.0%: 95% CI 93.18%–98.71%) tested seropositive, while 5 (3.0%; 95% CI 1.29%–6.82%) were seronegative. In the monovalent group, 158 (94.6%; 95% CI 90.08%–97.14%) were seropositive and 9 (5.4%; 95% CI 2.86%–9.92%) were seronegative.

The difference in overall seropositivity between groups was not statistically significant (*χ* ^2^ = 1.17; *p* = 0.28).

The correlation between time since vaccination and antibody titre was not significant for either formulation: monovalent HAV: *r* = 0.148; *p* = 0.057; bivalent HAV/HBV: *r* = −0.081; *p* = 0.302.

As antibody titres were not normally distributed, results are presented as GMCs. The geometric mean anti‐HAV IgG concentration was 6.3 IU/mL (95% CI 5.49–7.25) among monovalent vaccine recipients and 9.0 IU/mL (95% CI 8.01–10.26) among those vaccinated with the bivalent formulation, with a statistically significant difference between groups (*p* < 0.001). For descriptive purposes, arithmetic means (± SD) were 8.3 ± 4.6 IU/mL in the monovalent group and 10.9 ± 4.6 IU/mL in the bivalent group.

In the monovalent group, GMCs were 5.3 IU/mL (95% CI 4.18–6.76) in males and 7.1 IU/mL (95% CI 6.06–8.37) in females (*p* = 0.087). In the bivalent group, GMCs were 8.8 IU/mL (95% CI 7.18–10.68) in males and 9.3 IU/mL (95% CI 7.91–10.88) in females (*p* = 0.49).

Statistical comparison between the two vaccine groups revealed no significant differences for any of the variables examined: history of prior HAV serological testing (i.e., previous HAV serology performed before study enrolment, either for screening or occupational surveillance) (*p* = 0.94), contact with hepatitis A cases (*p* = 0.96), consumption of raw seafood products (*p* = 0.75) and travel to endemic areas (*p* = 0.68).

A multivariate linear regression model was used to identify independent predictors of antibody titre, including age, gender, vaccine formulation (A vs. A+B), years elapsed since the last vaccination and clinical–behavioural covariates. Age was the only variable independently associated with IgG titre (*β* = 0.32; 95% CI 0.10–0.43; *p* < 0.001). No significant associations were found for the remaining variables (gender, blood group, travel, HA contact, raw seafood consumption or previous serology) (Table 1).

**TABLE 1:** Multivariable linear regression model assessing independent determinants of IgG antibody titre.

Variable (category vs. reference)	*β* coefficient	95% CI	*p*
Age (per year increase)	+0.32	0.10–0.43	< 0.001
Vaccination group (A+B vs. A)	+0.73	−0.20 to 1.66	0.24
Gender (M vs. F)	−0.69	−1.71 to 0.33	0.18
Travel history (yes vs. no)	+0.21	−1.01 to 1.43	0.74
HA contact (yes vs. no)	+0.15	−1.12 to 1.48	0.82
Raw seafood consumption (yes vs. no)	−0.01	−1.05 to 1.04	0.99
Previous serology (yes vs. no)	−0.35	−1.65 to 0.94	0.59
Years since last vaccination	−0.11	−0.59 to 0.36	0.65

## 4 Discussion

This study demonstrates the long‐term persistence of HAV immunity more than two decades after vaccination. Protective antibody titers were maintained in nearly all participants (95.8%), confirming the durability of vaccine‐induced protection. These results are consistent with previous evidence [26] showing that hepatitis A vaccination induces sustained humoral immunity [27, 28] and contributes significantly to herd protection and the long‐term decline in HAV incidence [29, 30].

No significant differences in overall seroprotection were observed between recipients of the monovalent and bivalent HAV/HBV vaccines, indicating comparable long‐term immunogenicity. However, the higher mean IgG concentrations detected among bivalent vaccine recipients suggest a potential synergistic or adjuvant‐like effect between HAV and HBV antigens, consistent with prior observations of enhanced antibody responses following co‐administration of the two antigens [30, 31]. In addition to possible immunological benefits, the bivalent formulation offers practical advantages, facilitating higher vaccine uptake—particularly in resource‐limited or hard‐to‐reach populations [32, 33].

Gender‐related differences in vaccine response were observed only among recipients of the monovalent vaccine, where females exhibited higher mean IgG titers than males, although this difference approached but did not reach statistical significance.

The higher antibody titers observed among female participants are consistent with well‐established sex‐related differences in immune function [34, 35, 36]. Biological and hormonal factors, including oestrogen‐mediated enhancement of B‐cell activity and the dual expression of X‐linked immune genes such as *TLR7* and *CD40L*, contribute to stronger humoral and cellular responses in females [37, 38, 39]. These mechanisms not only enhance vaccine responsiveness but may also play a role in the greater longevity and reduced infectious mortality observed in women. Collectively, these findings highlight the importance of incorporating sex as a biological variable in vaccine immunogenicity research and long‐term immunity studies [38].

Limited data on combined HAV/HBV vaccines also suggest that female gender and younger age are associated with enhanced immunogenicity, underscoring the importance of considering gender as a biological variable in vaccine research.

Analysis of behavioural and exposure factors revealed no significant associations between antibody levels and variables such as travel to endemic areas, seafood consumption or occupational exposure. Interestingly, among bivalent vaccine recipients, those who had travelled to HAV‐endemic regions exhibited higher antibody titers (*p* = 0.03). The observed increase in antibody titers among travellers to endemic areas may reflect either subclinical exposure or, more plausibly, a recall of long‐term immune memory [40], as no serological evidence of recent infection was available.

Because the data on breakthrough infections were not available, no conclusions on vaccine effectiveness could be drawn and the results should be interpreted in terms of long‐term immunogenicity. Future studies including booster challenge could help assess immune memory.

No booster dose was administered to seronegative participants; therefore, the presence of immune memory could not be evaluated for them.

From a public health perspective, these findings reaffirm the long‐term immunogenicity of HAV vaccination and its central role in preventing infection and controlling outbreaks [41]. Maintaining epidemiological surveillance remains crucial to monitor population susceptibility, particularly among adults and travellers. Vaccination should be actively offered to all susceptible individuals and integrated opportunistically within other immunisation or certification programs to sustain high coverage and prevent re‐emergence [42]. Furthermore, the use of the combined HAV/HBV vaccine should be considered within occupational health strategies, particularly for HCWs, who are at risk of exposure to both viruses [43]. Integrating the combined formulation into pre‐employment or routine booster programs could enhance compliance, optimise resource utilisation and strengthen overall protection among this high‐priority group.

Comparable findings were reported in a Swiss survey of 6000 residents, in which HAV vaccination coverage among at‐risk individuals was low (20%–29%) and perceived risk similarly limited (21%–33%) [44]. Both studies highlight a persistent lack of awareness of HAV infection risk and vaccination status, even among well‐educated or health‐conscious populations.

The present study identified a significant difference in mean IgG titers between vaccine groups (*p* < 0.001), confirming stronger antibody responses among recipients of the bivalent HAV/HBV formulation. The homogeneity of the study population—characterised by good health, complete vaccination records and consistent follow‐up—likely reduced confounding, strengthening the reliability of this finding.

This study has limitations. The sample size was moderate and composed exclusively of healthcare workers, a generally healthy, homogeneous and occupationally exposed group—which may limit generalisability. Future research should include larger, demographically diverse cohorts encompassing different age groups, occupations and comorbidities to better define the long‐term kinetics of HAV antibodies and assess potential booster requirements.

Finally, sustained epidemiological surveillance and continued access to free vaccination for contacts of confirmed cases remain essential to prevent new outbreaks. Offering HAV vaccination to all susceptible adults at any vaccination encounter—whether for travel, certification or other immunisations—would help reduce the reservoir of susceptible individuals and minimise outbreak risk.

## 5 Conclusion

In 2016, the World Health Organization (WHO) launched a global strategy to eliminate viral hepatitis as a public health threat by 2030 [45]. Despite the availability of safe and effective HAV vaccines [30], outbreaks of acute hepatitis A continue to occur globally. Contributing factors such as climate change, globalisation, environmental shifts and population mobility complicate control efforts and highlight the need for robust surveillance systems.

This study contributes to that global objective by confirming that protective anti‐HAV antibodies persist for more than two decades after vaccination and that both monovalent and combined HAV/HBV vaccines provide comparable, long‐lasting protection. These results reinforce the importance of maintaining HAV vaccination within national immunisation programs.

Future research should aim to identify immunological correlates of durable protection, explore host–virus interactions influencing long‐term immunity and optimise vaccination strategies for diverse demographic and socio‐economic contexts [46]. Continued strengthening of vaccination policies and surveillance will be essential to achieving the WHO 2030 hepatitis elimination goals and preventing HAV resurgence worldwide.

## Conflicts of Interest

The authors declare no conflicts of interest.

## Data Availability

The data that support the findings of this study are available on request from the corresponding author. The data are not publicly available due to privacy or ethical restrictions.
